# Silicalite-Supported Ni Catalysts for Efficient CO_2_ Conversion into CH_4_

**DOI:** 10.3390/molecules31071215

**Published:** 2026-04-07

**Authors:** Nasir Shezad, Avik De, Ajaikumar Samikannu, Jyri-Pekka Mikkola, Farid Akhtar

**Affiliations:** 1 Division of Materials Science, Department of Engineering Science and Mathematics, Luleå University of Technology, 97187 Luleå, Sweden; nasir.shezad@ltu.se (N.S.); avik.de@associated.ltu.se (A.D.); 2 Technical Chemistry, Department of Chemistry, Chemical-Biological Centre, Umeå University, 90187 Umeå, Sweden; ajaikumar.samikannu@umu.se (A.S.); jyri-pekka.mikkola@umu.se (J.-P.M.); 3 Industrial Chemistry and Reaction Engineering, Johan Gadolin Process Chemistry Centre, Åbo Akademi University, FI20500 Åbo-Turku, Finland

**Keywords:** silicalite-1, nickel loading, CO_2_ methanation

## Abstract

The catalytic conversion of CO_2_ into methane (CH_4_) offers a sustainable solution to the worsening global warming scenario, especially for controlling CO_2_ levels. This study reports silicalite-1 supported Ni catalysts with different loadings for CO_2_ conversion to CH_4_, prepared via wet impregnation. The X-ray diffraction pattern revealed an increase in crystallite size at higher Ni loadings, which was further supported by N_2_ sorption, where the specific surface area and microporosity of the catalysts were decreased. There was a slight shift in the reducibility of the catalysts, potentially indicating the impact of loading on dispersion and spatial distribution. The catalyst performance was evaluated over a range of temperatures at 5 bar and a GHSV of 20,000 mL g_cat_^−1^ h^−1^. Surprisingly, the Ni(5)@Silicalite-1 exhibited higher CO_2_ conversion efficiency across the range of temperatures compared to Ni(10)@Silicalite-1. The NiO(5)@Silicalite-1 demonstrated a maximum CO_2_ conversion of 88% at 450 °C, which was approximately 14% higher than that of the catalyst with a 10 wt.% loading. Notably, the CH_4_ selectivity pattern was quite identical across the catalysts, underscoring that the reaction pathways were unaffected by the loadings. The higher performance of NiO(5)@Silicalite-1 could be ascribed to smaller NiO crystallites and improved textural properties.

## 1. Introduction

Excessive burning of fossil fuels has led to severe global warming, causing unusual climate changes worldwide. Greenhouse gas emissions, especially carbon dioxide (CO_2_), have reached a new high level of 422.5 ppm in the atmosphere, posing a pressing challenge to maintaining the 1.5 °C rise in temperature to limit global warming [1]. The need of the time is to accelerate the efforts toward carbon capture and utilization (CCU). The CCU techniques facilitate the conversion of CO_2_ into value-added chemicals, such as methane (CH_4_), methanol, and syngas, using renewable hydrogen. Beyond mitigating CO_2_ emissions, such processes also provide an effective pathway for hydrogen storage (Power-to-gas) and transport, with CH_4_ serving as a convenient energy carrier for diverse applications. Among the existing CCU technologies, catalytic conversion of CO_2_ into CH_4_ is a promising technique with mature industrial applications [2,3,4,5,6].

The catalytic conversion of CO_2_ is carried out using transition-metal and noble-metal catalysts under varying temperature and pressure conditions. Noble metal catalysts, such as ruthenium (Ru), palladium (Pd), and platinum (Pt), have been reported to transform CO_2_ into various products with stable performance and high efficiency [7,8,9]. However, economic concerns and the availability of noble metals limit their use for commercial applications [10]. Transition metals, such as nickel (Ni), manganese (Mn), and iron (Fe), have been reported to catalyze the conversion of CO_2_ into various products with appreciable efficiency [11,12,13]. Among them, Ni received much attention due to its higher activity, CH_4_ selectivity, and availability [14]. Notably, the highly exothermic CO_2_ methanation reaction is thermodynamically favorable at low temperatures; however, it is challenging to activate inert CO_2_ molecules, and the reaction typically proceeds at higher temperatures. Elevated temperatures lead to coking and sintering of the Ni-based catalyst, resulting in deactivation [15,16,17].

The characteristics of catalysts strongly depend on the support material [18]. Commonly used support materials include porous materials such as alumina (Al_2_O_3_), zeolite, silica (SiO_2_), MOFs, and carbon [19,20,21,22]. The Al_2_O_3_ has been extensively investigated as a support material due to its thermal and chemical stability. However, Ni can form Ni aluminate (NiAl_2_O_4_), which is challenging to reduce and can significantly limit the catalyst’s performance [23]. The SiO_2_-based material has been employed due to its higher thermal stability and its ordered meso-structured nature. Chen et al. [24] investigated the cage-type properties of SBA-15 (mesoporous SiO_2_) to control the size of Ni nanoparticles by incorporating carboxylic functional groups (-COOH) into the pores. The modified SBA-15 facilitated tuning of Ni nanoparticle size, resulting in enhanced CO_2_ methanation performance and high CH_4_ selectivity.

Additionally, zeolites are microporous materials characterized by high surface areas, flexible pore chemistry, and thermal and chemical stability. The characteristics of zeolite-supported Ni catalysts depend on the nature of the support, porosity, and chemistry of the framework [25,26]. Yan et al. [27] investigated the impact of zeolite support on the activity of Ni catalysts for CO_2_ methanation. It was observed that the zeolite 13X-supported catalyst performs better than others due to the availability of Ni on the surface compared to confined nanoparticles in the ZSM-5 and BEA zeolites. Also, the chemistry of the zeolite framework significantly influences its surface properties, particularly CO_2_ adsorption, hydrothermal stability, and hydrophilicity [28]. The hydrothermal stability of the support material is crucial, as the CO_2_ methanation reaction produces water. At high temperatures, zeolite steaming leads to dealumination, rendering the catalyst unstable [29]. Interestingly, the hydrothermal stability of the zeolite can be tuned by increasing the Si/Al ratio. For example, Faujasite (Zeolite 13X, Zeolite Y) exhibits better hydrothermally stability in the form of zeolite Y compared to X, due to a higher Si/Al ratio [29]. Changes in the Si/Al ratio affect catalyst properties, particularly porosity, CO_2_ adsorption, acidity, and water affinity. CO_2_ adsorption may be altered by increasing the Si/Al ratio, due to changes in exchangeable cations and the framework structure. Bacariza et al. [30] studied the impact of the Si/Al ratio on zeolite Y-supported Ni catalysts for CO_2_ hydrogenation to CH_4_. The catalyst with a high Si/Al ratio exhibited higher CO_2_ conversion than the one with a lower ratio. The enhanced performance correlated with increased hydrophobicity, leading to rapid water removal from the catalyst’s surface and shifting the equilibrium toward the product. The removal of Al also leads to the development of stronger metal–support interactions, shifting the reduction temperatures of the catalysts to higher values [30]. Additionally, zeolites without Al in the framework, known as silicalite, are porous materials with high surface area and reduced affinity for water. Limited studies are exploring the silicalite for CO_2_ methanation. Herein, we investigated silicalite-1-supported catalysts with varying Ni loadings prepared by wet impregnation. The prepared catalysts were characterized using X-ray diffraction (XRD), scanning electron microscopy (SEM), energy-dispersive X-ray spectroscopy (EDX), temperature-programmed desorption (CO_2_-TPD), temperature-programmed reduction (H_2_-TPR), and N_2_ sorption isotherms. The catalysts were evaluated for CO_2_ methanation at various temperatures and 5 bar pressure, with a GHSV of 20,000 mL g_cat_^−1^ h^−1^. The Ni(5)@Silicalite-1 catalysts exhibited higher catalytic performance compared to the catalyst with higher Ni loading. The activity of the catalyst was correlated with better textural properties and smaller Ni nanoparticles, resulting in higher CO_2_ methanation at various temperatures.

## 2. Results

**Figure 1:**
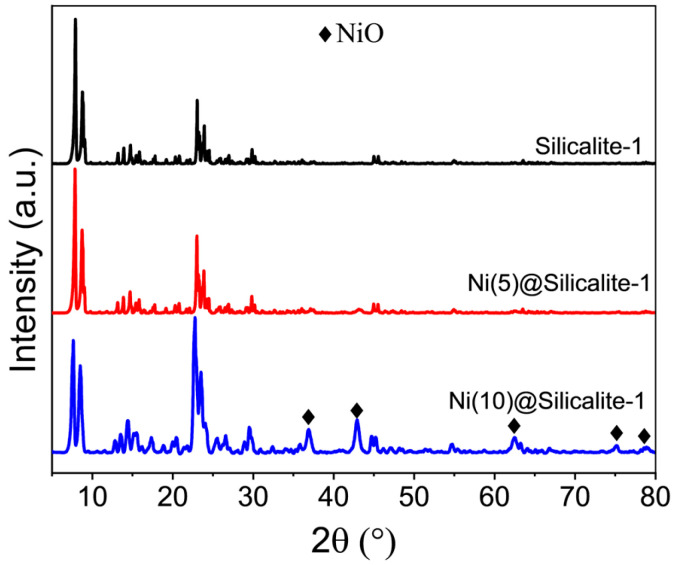
Crystallinity of the catalysts.

The powder X-ray diffraction (XRD) patterns (Figure 1) of pristine silicalite-1 and the Ni-loaded catalysts confirm that all samples are highly crystalline in nature. The silicalite-1 sample displays well-defined diffraction peaks corresponding to various crystallographic planes of the MFI-type zeolite structure, confirming its phase purity. Both Ni(5)@Silicalite-1 and Ni(10)@Silicalite-1 exhibit the characteristic peaks of silicalite, indicating that the structural framework of the host material remains intact upon Ni incorporation. In the case of Ni(10)@Silicalite-1 catalyst, additional diffraction peaks appear at 2θ values of approximately 37°, 43°, 62°, 75°, and 79°, which are consistent with the (111), (200), (220), (311), and (222) planes of NiO, respectively, confirming the formation of NiO crystallites [31]. For Ni(5)@Silicalite-1, only broad humps are observed in the same 2θ regions, suggesting the presence of highly dispersed, ultra-small NiO nanoparticles with limited crystallinity [32,33]. The gradual broadening of the silicalite peaks with increasing Ni content further supports partial surface coverage by NiO nanoparticles and suggests the possible introduction of lattice strain or slight structural disorder during impregnation and calcination.

**Figure 2:**
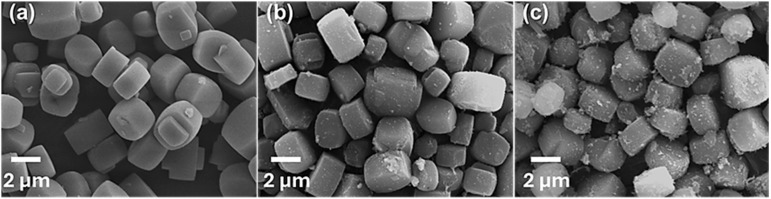
Morphology of the catalysts: (**a**) Silicalite-1, (**b**) Ni(5)@Silicalite-1, and (**c**) Ni(10)@Silicalite-1.

To investigate the morphological changes induced by NiO loading, scanning electron microscopy (SEM) analysis was performed. As shown in Figure 2, the pristine silicalite-1 sample (Figure 2a) exhibits well-defined hexagonal prism-shaped crystals with slightly rounded edges and smooth surfaces. The average crystal size is approximately 5 µm. Notably, intergrowth twin crystals are observed emerging from the hexagonal faces, suggesting a high degree of crystallinity and uniformity. Upon NiO impregnation, a progressive alteration in surface morphology is evident. The prismatic structure of the silicalite is retained in both Ni(5)@Silicalite-1 (Figure 2b) and Ni(10)@Silicalite-1 (Figure 2c), but surfaces become increasingly rough with higher Ni loading. In the case of Ni(5)@Silicalite-1, small NiO nanoparticles appear as discrete, pimple-like protrusions on the otherwise smooth silicalite surface, indicating uniform dispersion with minimal aggregation. Meanwhile, Ni(10)@Silicalite-1 displays a significantly rougher surface with widespread deposition and agglomeration of NiO particles. Excess NiO results in uneven surface coverage and visible nanoparticle clusters, suggesting oversaturation of the silicalite-1 surface.

**Figure 3:**
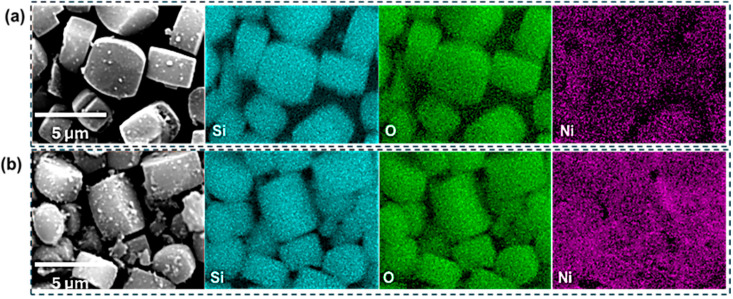
SEM-EDS maps of the catalysts: (**a**) Ni(5)@Silicalite-1 and (**b**) Ni(10)@Silicalite-1.

Energy-dispersive X-ray spectroscopy (EDS) mapping was performed in conjunction with SEM to further support the successful incorporation of NiO into the silicalite-1 framework, as shown in Figure 3. The Ni signal intensity is significantly higher in the Ni(10)@Silicalite-1 sample compared to Ni(5)@Silicalite-1, consistent with the increased Ni loading. While Ni is uniformly distributed across both samples, Ni(10)@Silicalite exhibits slightly enhanced signals at the edges of the silicalite crystals, suggesting localized surface deposition of NiO.

**Figure 4:**
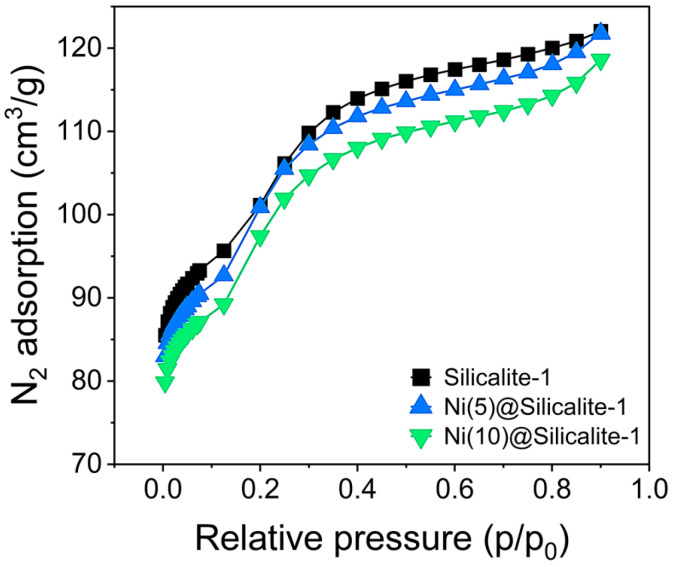
N_2_ sorption isotherms of the catalysts.

The textural properties of the pristine silicalite-1 and the catalysts were characterized by the N_2_ sorption isotherms, as shown in Figure 4 and Table 1. Both the support and catalyst samples exhibited similar sorption patterns, consistent with the characteristics of micro- and mesoporous materials. The steep N_2_ sorption at *p*/*p*_0_ < 0.15 bar shows the presence of a typical microporous structure, whereas the inflection point starting from the *p*/*p*_0_ > 0.15 bar indicates the existence of mesopores [34]. Loading of NiO obviously resulted in a decrease in surface area; more specifically, higher loading led to a greater decrease. The external and micropore area estimated from t-plots shows that NiO impregnation resulted in a loss of micropore area. Notably, there is an increase in the external surface area at the cost of a reduction in the micropore area. Importantly, at higher Ni loading, microporosity loss is more pronounced, suggesting pore blockage [35]. Furthermore, the loss of surface area with loading can be attributed to the dispersion of NiO. Typically, poorly dispersed Ni results in greater surface area blockage. Specifically, the Ni(5)@Silicalite-1 shows only a slight reduction in surface area compared to pristine silicalite, indicating that the mesopores remain largely accessible and unblocked. However, for Ni(10)@Silicalite-1, the substantial surface coverage and particle aggregation lead to a marked decrease in BET surface area, indicating pore blockage and reduced accessibility. The observed differences support the SEM surface morphology results and the XRD NiO crystallite size results, which reveal severe agglomeration of Ni on the support surface at higher loadings. Overall, a decrease in microporosity accompanied by an increase in mesoporosity indicated that the silicalite structure had further deteriorated as a result of the NiO precursor during the impregnation and calcination process [20,36].

**Figure 5:**
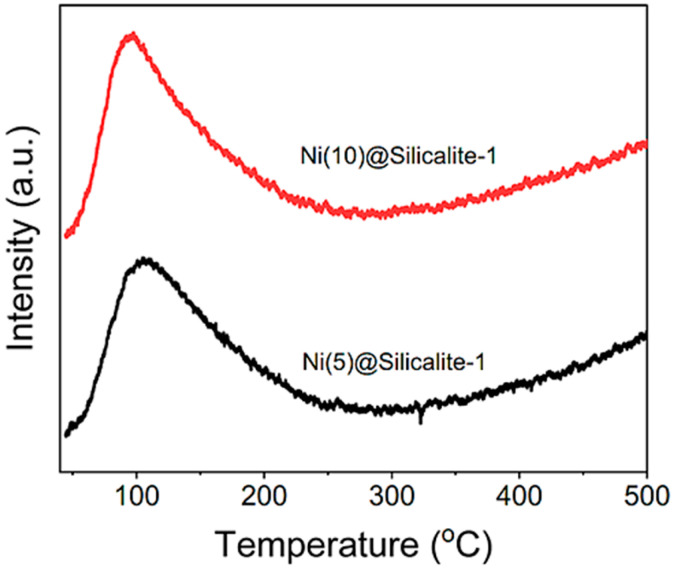
Basicity of the catalysts using CO_2_-TPD analysis.

The catalysts were further analyzed by CO_2_-TPD to estimate their basicity. The CO_2_ methanation process involves adsorbing CO_2_ on the catalyst’s surface, followed by activation and conversion to CH_4_. The adsorption sites are categorized into weak, medium, and strong basic sites, depending on the temperature required to desorb CO_2_ from the catalyst [37]. The silicalite support contains various hydroxyl groups, and CO_2_ might coordinate with them as carbonates and bicarbonates [37]. As shown in Figure 5, both catalysts exhibited a broad peak at temperatures below 250 °C, indicating CO_2_ desorption from weak-to-medium basic sites. The peak pattern is almost identical in both catalysts, underscoring that the desorption might originate from chemisorption and physisorption sites that are identical [21,38]. The peak intensity is relatively higher in Ni(10)@Silicalite-1, which can be attributed to the higher NiO loadings. Calculated amount of desorbed CO_2_ was 0.44 and 0.046 mmol/g for Ni(5)@Silicalite-1 and Ni(10)@Silicalite-1, respectively. The impregnated NiO contributes to CO_2_ adsorption as carbonates and bicarbonates via O^2−^ lattice oxygen and Ni^2+^ cation sites. There is no peak at higher temperatures, indicating the absence of strong basic sites. During high-temperature reactions, CO_2_ may adsorb and activate as formate species directly on the Ni-NiO interface, depending on the availability and dispersion of Ni species [39]. Westermann et al. [40] reported the absence of the CO_2_-TPD peak at higher temperatures for the 5% Ni/USY catalyst due to a lower amount of CO_2_, which might not rise to a peak by the TCD, whereas the catalyst with higher loadings ≥ 10% showed a clear peak in the medium-temperature range. This also indicates that silicalite lacks strong basic sites [41] and chemisorbed CO_2_ mainly depends on NiO loadings.

**Figure 6:**
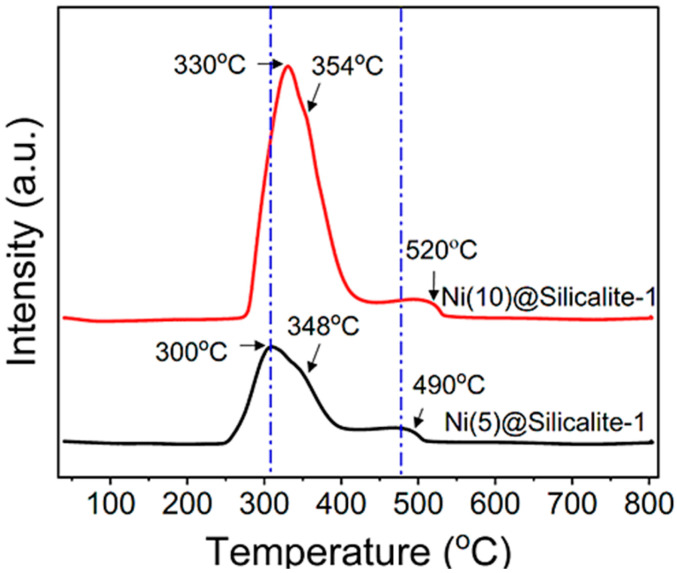
Reduction profile of the catalyst by H_2_-TPR analysis.

To trigger the CO_2_ methanation reaction, it is essential to activate H_2_ into ^•^H, which depends on the availability of Ni^0^ generated by NiO reduction. H_2_-TPR analysis was performed to estimate the catalysts’ reduction profiles. The reduction temperature and peak patterns by the analysis also provide information about the spatial distribution and strength of Ni attachment to the support [33,42]. As shown in Figure 6, both catalysts exhibited identical behavior, displaying three distinct peaks with varying intensities. The peak intensities may be correlated with the Ni content; clearly, the catalyst with 10% Ni shows higher intensity. The peaks at 300 °C and 330 °C represent the weakly associated surface-bound NiO nanoparticles, whereas the small peak at 490–520 °C indicates the strongly attached nanoparticles with strong metal–support interaction [43,44]. Another main peak, at approximately 350 °C, indicates the medium strength of NiO attachment on the catalyst surface. As revealed by N_2_ sorption, the catalyst contains mesopores that can confine NiO within the pores of the silicalite support. The minor peaks in the 490–520 °C range could represent confined NiO, resulting in different spatial distributions and metal–support interactions. A shift in the positions of the TPR peaks can be attributed to the strength of metal–support interactions across the catalysts.

## 3. Catalytic Activity

**Figure 7:**
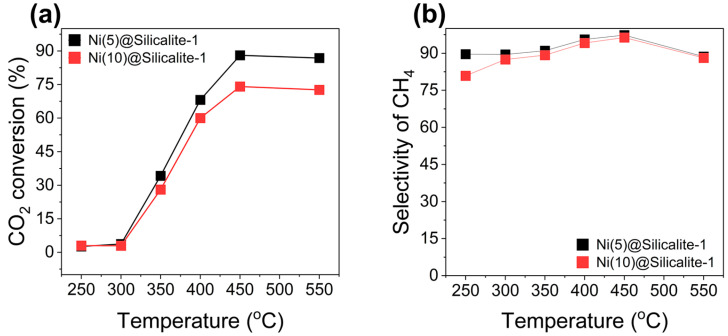
Catalytic activity of the catalyst at different temperatures: (**a**) CO_2_ conversion, (**b**) CH_4_ selectivity.

The catalytic activity of the catalysts was evaluated for CO_2_ conversion into CH_4_ across a range of temperatures at 5 bar pressure and a GHSV of 20,000 mL g_cat_^−1^ h^−1^. Thermodynamically, the CO_2_ conversion reaction is exothermic, and therefore, it exhibits higher conversion at lower temperatures. Therefore, optimized temperature conditions are essential for achieving the catalyst’s maximum performance. As shown in Figure 7a, both catalysts exhibited increased activity with increasing temperature. This indicates that CO_2_ conversion is kinetically limited at low temperatures but proceeds at appreciable rates as temperature increases [45,46]. More specifically, performance was quite low at 250 °C, and the difference in activity was negligible, indicating the intrinsic limitation of the Ni-based catalyst, which performs better at elevated temperature conditions. The Ni(5)@Silicalite-1 catalyst exhibited better performance than the Ni(10)@Silicalite-1 catalyst, with a maximum CO_2_ conversion of approximately 88% at 450 °C, which is 14% higher than that of the latter. The difference in conversion can be correlated with the catalyst’s characteristics, such as surface area, reducibility, and the size of NiO nanoparticles. As revealed by N_2_ sorption, surface area and micropore area decreased at higher loading, negatively affecting the catalyst’s catalytic activity. The performance of both catalysts decreased after 450 °C due to associated thermodynamic limitations. As CO_2_ methanation releases heat, high temperature is not favorable.

In addition, the selectivity of CH_4_ was evaluated over temperature, and the results are shown in Figure 7b. The selectivity of CH_4_ followed a similar trend for both the catalysts, with marginally higher values for Ni(5)@Silicalite. Like CO_2_ conversion, selectivity of CH_4_ was highest at 450 °C with values of 97% and 96% over Ni(5)@Silicalite and Ni(10)@Silicalite, respectively. A further increase in temperature led to a decrease in CH_4_ selectivity from 97% to 88%, underscoring that high temperatures negatively impacted CH_4_ formation. The decrease in CH_4_ selectivity is related to the exothermic nature of methanation (CO_2_ + 4H_2_ → CH_4_ + 2H_2_O) reaction and the endothermic nature of the reverse water gas shift reaction (RWGS, CO_2_ + H_2_ → CO + H_2_O) [47,48]. The RWGS began to dominate at higher temperature conditions due to its thermodynamics, thereby promoting the formation of CO, which is otherwise a side product of the CO_2_ methanation reaction. Notably, the formation of a specific product is influenced by catalyst type, process conditions, and reaction mechanism. The Ni has been reported to be selective towards CH_4_ formation. However, there are studies in which CO has been reported to increase depending on the reaction mechanism. In this study, no CO_2_ desorption peak was observed at high temperatures in the CO_2_-TPD analysis, indicating that adsorption and activation of CO_2_ occurred at the surface of Ni^0^. Formation of formate species through an associated mechanism might be dominating where CO_2_ molecules react with ^•^H on the surface of the Ni^0^ (CO_2_^−^ + H→HCOO^−^) [49]. The higher selectivity of CH_4_ over both catalysts indicated that formate intermediates were hydrogenated to a greater extent. Westermann et al. [40] examined the mechanism of CO_2_ methanation over a Ni/USY catalyst and found that the formate ion concentration rapidly decreased with a rise in temperature compared to ^•^CO formation, indicating the complete hydrogenation of formate species into CH_4_. Thus, a Ni loading of 5 wt.% over silicalite-1 proved to be optimal for efficient CO_2_ conversion into CH_4_ due to the textural properties and size of the catalyst’s nanoparticles.

## 4. Materials and Methods

### 4.1. Chemicals

The nickel(II) nitrate hexahydrate (99.999%) was purchased from Sigma Aldrich (St. Louis, MO, USA) and used without further purification. Ethanol (absolute, ≥99.5%) was procured from Merck (Darmstadt, Germany).

### 4.2. Preparation of Ni@Silicalite-1

Two catalyst samples were prepared by varying the amount of Ni-salt relative to silicalite using the wet impregnation method. Initially, the specified amount of nickel(II) nitrate hexahydrate was dissolved in 50 mL of ethanol in a 250 mL beaker under stirring using a magnetic stirrer over a hot plate. Meanwhile, silicalite-1 was degassed overnight at 200 °C under vacuum. Subsequently, 1 g of the degassed silicalite-1 powder was added to the nickel solution. The mixture was stirred for 1 h at 20 °C, followed by 1 h at 30 °C, and then at 50 °C until the solvent had evaporated entirely. The resulting solid was then oven-dried at 80 °C overnight. Here, Ni-containing silicalite-1 samples were synthesized by impregnating 1 g of silicalite-1 with 248 mg and 496 mg of nickel(II) nitrate hexahydrate, corresponding to calculated Ni loadings of approximately 5 wt.% and 10 wt.%, respectively. The catalyst samples were first placed in a vacuum oven and heated overnight at 200 °C. Subsequently, the samples were calcined in a muffle furnace at 500 °C for 4 h, with a heating ramp rate of 1 °C/min, and were designated as Ni(5)@Silicalite-1 and Ni(10)@Silicalite-1, respectively. To validate the Ni contents, the catalysts were analyzed using ICP-MS, and the results showed 4.7 wt.% and 9.9 wt.% Ni, corresponding to 5 wt.% and 10 wt.% loading in Ni(5)@Silicalite-1 and Ni(10)@Silicalite-1 samples, respectively.

### 4.3. Characterization

The physical and chemical characteristics of the synthesized catalysts were analyzed using various analytical techniques. The X-ray diffraction data of the catalysts and silicalite were collected through the X-ray diffractometer using Cu k-α radiation, λ = 1.5418 Å (Empyrean, Malvern Panalytical, Malvern, UK) and processed using Highscore Plus (Version 5.2, Malvern Panalytical) software. The morphology of the catalysts was analyzed using scanning electron microscopy (SEM, JSM-IT300LV, JEOL GmbH, Freising, Germany) with an applied voltage of 10 kV and probe current 30 n, using a secondary electron detector (SED). Before the SEM investigation, a 10 nm platinum coating was applied to the sample surfaces using a sputter coater (Leica EM ACE200, Leica Microsystems, Wetzlar, Germany). Elemental distribution mapping of catalysts was obtained using energy-dispersive X-ray spectroscopy (EDS) with SEM. The Oxford X-Max 80 EDS detector (Oxford Instruments plc, Abingdon, UK) was used, and the AZTEC software package (Version 5.1) was used to record and process the EDS data. During EDS scans, the applied voltage and probe current increased to 20 kV and 50 nA, respectively. The H_2_-TPR and CO_2_-TPD experiments were conducted using a calibrated BELCAT II instrument (MicrotracBEL Corp., Osaka, Japan) equipped with a thermal conductivity detector (TCD). Typically, 50–60 mg of pre-calcined sample was loaded into a quartz U-shaped reactor, pretreated at 400 °C for 1 h under an argon atmosphere, and then cooled to 40 °C. The reducibility profile was recorded using 10% H_2_/Ar up to 800 °C with a heating rate of 10 °C/min. For CO_2_-TPD measurements, approximately 50–60 mg of each sample was in situ pretreated at 400 °C under He gas for 1 h, then cooled to 40 °C. Subsequently, the catalyst was saturated with 10% CO_2_/He for 2 h at 40 °C. Subsequently, the sample was flushed with helium for 30 min to remove traces of physiosorbed CO_2_. The desorption of chemisorbed CO_2_ was carried out from 40 °C to 500 °C at a heating rate of 10 °C/min under a He gas flow. After each measurement, the amount of chemisorbed CO_2_ was determined from a calibration curve obtained by varying the CO_2_ volume in He. The N_2_ adsorption isotherms were recorded at −196.15 °C (77 K) after degassing the samples at 219.85 °C (493 K) for 3 h using Gemini VII 2390, Micromeritics, Norcross, GA, USA. The surface areas were calculated using the BET method, and the micropore and external surface areas were estimated from t-plots.

### 4.4. Catalytic Measurements

The performance of the synthesized catalysts was evaluated in a continuous fixed-bed reactor system for CO_2_ conversion to CH_4_. The reactor setup consists of a quartz glass tube enclosed within a stainless-steel tube, which is positioned inside the tube furnace. A 300 mg catalyst sample was mixed with 1000 mg of quartz sand (Sigma Aldrich, 50–70 mesh) and loaded into a quartz glass tube, supported by glass wool at both the bottom and the top. After closing the reactor, a leak test was performed at 10 bar to ensure no gas leakage. The reactor temperature was increased to 500 °C at a heating rate of 10 °C/min, and the catalyst was reduced under 20% H_2_/80% N_2_ atmosphere for 1 h. The reactor was cooled to 250 °C, and the pressure was adjusted to 5 bar. Then, 100 mL of reaction gas mixture (CO_2_/H_2_/N_2_) with a molar ratio of 12:48:40 was introduced at a GHSV of 20,000 mL g_cat_^−1^ h^−1^, and catalytic experiments were performed at different temperatures from 250 to 500 °C. The reactor outlet was connected to the GC-TCD/FID for analysis of the product gases. The CO_2_ conversion and CH_4_ selectivity were evaluated using the following Equations (1)–(3), where F represents the molar flow of the respective gas at the inlet and outlet of the reactor.

(1) $${\mathrm{CO}}_{2\;}\;\mathrm{Conversion},\;X_{{\mathrm{CO}}_{2}}\;(\%)=\frac{F_{{{\mathrm{CO}}_{2}}_{\mathrm{in}}}-{\;F}_{{{\mathrm{CO}}_{2}}_{\mathrm{out}}}}{F_{{{\mathrm{CO}}_{2}}_{\mathrm{in}}}}\times 100$$

(2) $${CH}_{4}\;Selectivity,\;S_{{CH}_{4}}\left(\%\right)=\frac{F_{{{\mathrm{CH}}_{4}}_{\mathrm{out}}}}{F_{{{\mathrm{CH}}_{4}}_{\mathrm{out}}}+F_{{CO}_{out}}}\times 100$$

(3) $$CO\;Selectivity,\;S_{CO}\left(\%\right)=\frac{F_{{CO}_{out}}}{F_{{{\mathrm{CH}}_{4}}_{\mathrm{out}}}+F_{{CO}_{out}}}\times 100$$

## 5. Conclusions

This work reports the evaluation of silicalite-1-supported Ni catalysts with varying Ni loadings, prepared by wet impregnation for CO_2_ methanation. The higher loading resulted in poorly dispersed Ni species, as evidenced by the increased crystallite size revealed by XRD analysis. The textural properties of the silicalite-1 support were severely affected, resulting in a reduction in micropore area for catalysts with higher Ni contents. A slight variation in the reduction temperatures of the catalysts, as revealed by H_2_-TPR, indicated changes in the metal–support interaction and dispersion of Ni, further supporting the XRD results. The catalytic CO_2_ conversion performance of Ni(5)@Silicalite-1 was superior to that of Ni(10)@Silicalite-1, with a maximum conversion of 88% at 450 °C under 5 bar and a GHSV of 20,000 mL g_cat_^−1^ h^−1^. Although Ni(5)@Silicalite-1 exhibited 14% higher CO_2_ conversion than Ni(10)@Silicalite-1 catalyst, the CH_4_ selectivity was approximately identical over both catalysts across the temperatures. The enhanced performance of the catalyst with lower loading can be attributed to its better textural properties and smaller Ni nanoparticle crystallite size.

## Figures and Tables

**Table 1:** Textural properties of the catalysts.

Catalyst	S_total_ (m^2^/g)	S_ext_ (m^2^/g)	S_micro_ (m^2^/g)
Silicalite-1	381	194	187
Ni(5)@Silicalite-1	371	241	129
Ni(10)@Silicalite-1	357	239	117

## Data Availability

The data presented in this study are available on request from the corresponding author.
